# Alterations of Central Liver Metabolism of Pediatric Patients with Non-Alcoholic Fatty Liver Disease

**DOI:** 10.3390/ijms231911072

**Published:** 2022-09-21

**Authors:** Nikolaus Berndt, Christian A. Hudert, Johannes Eckstein, Christoph Loddenkemper, Stephan Henning, Philip Bufler, David Meierhofer, Ingolf Sack, Susanna Wiegand, Iwona Wallach, Hermann-Georg Holzhütter

**Affiliations:** 1 Institute of Computer-Assisted Cardiovascular Medicine, Charité-Universitätsmedizin Berlin, Corporate Member of Freie Universität Berlin and Humboldt-Universität zu Berlin, 13353 Berlin, Germany; 2 Department of Pediatric Gastroenterology, Nephrology and Metabolic Diseases, Charité-Universitätsmedizin Berlin, Corporate Member of Freie Universität Berlin and Humboldt-Universität zu Berlin, 13353 Berlin, Germany; 3 Institute of Biochemistry, Charité-Universitätsmedizin Berlin, Corporate Member of Freie Universität Berlin and Humboldt-Universität zu Berlin, 10117 Berlin, Germany; 4 Gemeinschaftspraxis fuer Pathologie Pathotres, 12247 Berlin, Germany; 5 Mass Spectrometry Facility, Max Planck Institute for Molecular Genetics, 14195 Berlin, Germany; 6 Department of Radiology, Charité-Universitätsmedizin Berlin, Corporate Member of Freie Universität Berlin and Humboldt-Universität zu Berlin, 10117 Berlin, Germany; 7 Center for Chronically Sick Children, Charité-Universitätsmedizin Berlin, Corporate Member of Freie Universität Berlin and Humboldt-Universität zu Berlin, 13353 Berlin, Germany

**Keywords:** mathematical modeling, liver tissue, histology, proteomics, plasma profile

## Abstract

Non-alcoholic fatty liver disease (NAFLD) is the most common chronic liver disease in children and is associated with overweight and insulin resistance (IR). Almost nothing is known about in vivo alterations of liver metabolism in NAFLD, especially in the early stages of non-alcoholic steatohepatitis (NASH). Here, we used a complex mathematical model of liver metabolism to quantify the central hepatic metabolic functions of 71 children with biopsy-proven NAFLD. For each patient, a personalized model variant was generated based on enzyme abundances determined by mass spectroscopy. Our analysis revealed statistically significant alterations in the hepatic carbohydrate, lipid, and ammonia metabolism, which increased with the degree of obesity and severity of NAFLD. Histologic features of NASH and IR displayed opposing associations with changes in carbohydrate and lipid metabolism but synergistically decreased urea synthesis in favor of the increased release of glutamine, a driver of liver fibrosis. Taken together, our study reveals already significant alterations in the NASH liver of pediatric patients, which, however, are differently modulated by the simultaneous presence of IR.

## 1. Introduction

Non-alcoholic liver disease (NAFLD) is an umbrella term that refers to a broad spectrum of chronic liver pathologies, which are closely associated with obesity and insulin resistance (IR) but are not primarily caused by an excessive intake of alcohol, bacterial or viral infection, liver intoxication by medical drugs or xenobiotics, or an autoimmune response of the liver. NAFLD ranges from simple liver steatosis, characterized by a macro-vesicular accumulation of fat in at least 5% of hepatocytes, to non-alcoholic steatohepatitis (NASH), a latent inflammation of the liver that can progress to more serious disease stages, such as advanced fibrosis, cirrhosis, liver failure, and hepatocellular carcinoma [1,2,3].

With the increasing prevalence of severe obesity in children and adolescents in high-income countries, we now witness a dramatic increase in NAFLD prevalence in this age group [2]. In a large European cohort of 16,390 overweight or obese children and adolescents, elevated liver transaminases were present in 11% of the study population [1]. In an autopsy study of 742 children, NAFLD was present in 9.6% of all reviewed autopsy reports, but the prevalence increased to 42% in obese subjects. In total, 23% of the children with fatty liver showed signs of NASH [3]. Within the next 10 years, NASH is expected to become the most prevalent cause of liver pathology, liver failure, and indication for liver transplantation in the Western world [4].

NAFLD and type 2 diabetes (T2DM) often coexist. The prevalence of NAFLD is about 60% in adult patients with T2DM [5]. In a pediatric multicenter study, nearly 30% of 675 children with NAFLD either had prediabetic glucose metabolism or manifested T2DM. Prevalence of NASH was doubled (43.2% vs. 22%) in children with T2DM as compared to children with unaltered glucose metabolism [6].

Etiologically, the relationship between NAFLD and T2DM is bidirectional [7,8]. Diabetes promotes the progression of NAFLD to NASH and increases the risk of cirrhosis and hepatocellular carcinoma. On the other hand, NAFLD is associated with an increased risk of developing T2DM. Although NAFLD and diabetes represent two sides of the same coin [9], the associated pathophysiological changes in the liver can be quite different. While increased insulin levels in the case of IR increase the expression of hepatic glucokinase and thus the glucose uptake capacity of the liver [10], pro-inflammatory cytokines such as interleukin 6 cause decreased expression of hepatic glucokinase [11]. Thus, when studying metabolic changes in the liver during the progression of NAFLD, one must include the possible contribution of elevated insulin levels due to systemic IR.

While histological assessment of liver structure based on liver biopsies is still the gold standard of NASH diagnosis [12], currently used clinical markers do not allow for the evaluation of metabolic liver functions that ultimately determine the severity of the disease. Commonly observed changes in the metabolic plasma profile of juvenile NASH patients [13], such as elevated levels of fasting glucose, total triacylglycerol (TAG), and total cholesterol, mirror only faintly liver metabolism due to the involvement of other organs, in particular peripheral adipose tissue. Measurements of metabolite concentrations and enzyme fluxes in hepatocytes isolated from a NASH liver can hardly recapitulate the in vivo situation. Except for hepatic TAG accumulation, which can be easily assessed by ultrasound magnetic resonance imaging and liver biopsy, there is virtually nothing known about metabolic adaptations at the cellular level during NASH progression. To overcome this unsatisfactory situation, we applied a computational approach based on a physiology-based, validated, mathematical model of hepatic metabolism [14], which we extended by a molecular resolved model of the hepatic lipid droplet turnover [15]. The model was parameterized in a patient-specific manner based on protein-intensity profiles of metabolic enzymes measured in biopsy samples. We assessed the central metabolic capacities of the liver in a pediatric cohort of obese patients with biopsy-proven NAFLD and investigated the relationship between individual metabolic alterations of hepatic metabolism and the degree of NASH activity (as defined by the grade of inflammation and fibrosis) and IR.

## 2. Results

### 2.1. Patients’ Characteristics

A total of 71 overweight children and adolescents (16 girls, 55 boys) aged between 10–17 years were included in this study. Plasma markers indicating the presence of systemic inflammation, injury of hepatocytes, hyperglycemia, IR, and dyslipidemia were determined (Table 1). Overall, the cohort was characterized by the presence of hypertriglyceridemia (136 mg/dL versus the age-matched normal value of 90 mg/dL [16]) and IR (HOMA-IR (homeostatic model assessment for insulin resistance) = 7 versus normal values of 1.7 and 1.4 in 11-year-old girls and boys [17]). The cohort-mean value for total plasma cholesterol was close to the age-matched normal value of about 160 mg/dL but seven patients had values larger than 200 mg/dL indicative of hypercholesterolemia [16]. Taking values of HOMA-IR greater than 5 as indicative of pre-existing T2DM [18], 68% of our pediatric cohort were pre-diabetic. Of note, two patients were clinically diagnosed with T2DM, while one patient had insulin-dependent T1DM.

A comprehensive histological analysis of the biopsy samples was carried out by experienced pathologists, according to the NASH clinical research network criteria (Table 2). A broad spectrum of NAFLD disease activity was found within our patient cohort from simple steatosis to severe NASH. Staging of fibrosis ranged from F0 (no fibrosis) to F3 (advanced fibrosis) in approximately equal distribution, while cirrhosis (F4) was not present. Inflammatory activity was found in 45 (63.4%) or 49 patients (69%) in zone 1 (portal inflammation) or zone 3 (lobular inflammation), respectively. Ballooning of hepatocytes, an indication of severe hepatocellular damage and cytoskeletal injury in NASH [19], was observed in 34 patients (48%).

### 2.2. Computational Assessment of Metabolic Functions and Maximal Metabolic Capacities

Based on proteomics data, we assessed the metabolic performance of the liver using an extended version of HEPATOKIN1 (see Section 4). We used two different metabolic measures: Metabolic functions were defined by the maximal flux (or maximal concentration) obtained during a standardized 24 h plasma profile of nutrients and hormones (see Supplemental Figure S1). Maximal metabolic capacities were assessed as maximal flux values or concentrations at a steady state obtained under physiological conditions (see Section 4).

**Figure 1:**
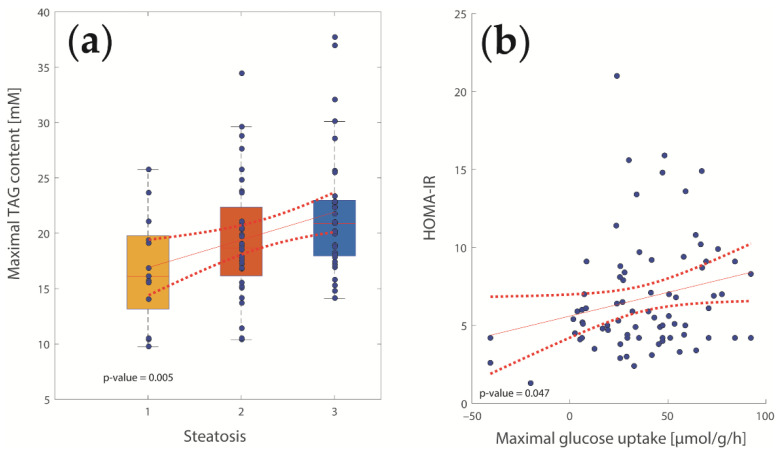
Model validation. (**a**) Relationship between hepatic triacylglycerol (TAG) content and histologically assessed grade of steatosis; (**b**) Relationship between homeostatic model assessment for insulin resistance (HOMAR-IR) and hepatic glucose uptake.

For validation, we looked at those computed metabolic functions that could be directly compared with clinical data. Hepatic TAG content was the only metabolic function that was directly determined experimentally by histology (grading between 0 and 3). As shown in Figure 1a, the calculated TAG content was significantly associated with the histologically assessed degree of steatosis (*p* = 0.005). Control analysis revealed that accumulation of TAG was due to perilipin 2-dependent prevention of TAG lipolysis from lipid droplets, while uptake of fatty acids, TAG-synthesis, and β-oxidation were not significantly altered. A second clinical parameter suited for comparison with model results was HOMA-IR, a marker of systemic IR [18]. In the early phase of T2DM, the pancreatic β-cells respond to elevated plasma glucose levels with increased insulin production. Insulin stimulates the expression of the enzyme glucokinase [10,20] and thus improves the glucose uptake capacity of the liver. The correlation analysis shown in Figure 1b revealed a positive association between HOMA-IR and the computed hepatic glucose uptake (*p* = 0.047), thus confirming the relationship between IR and hepatic glucose metabolism.

**Figure 2:**
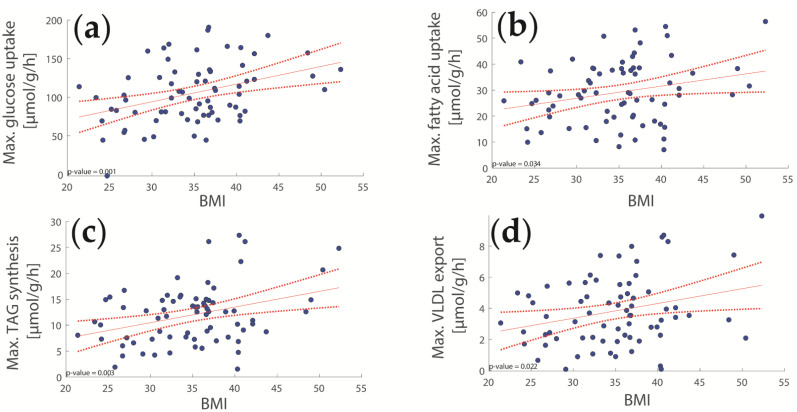
Relationship between the body mass index (BMI) and hepatic metabolic functions. (**a**) Glycolysis; (**b**) Fatty acid uptake; (**c**) TAG synthesis; (**d**) Lipoprotein synthesis.

In adults, the BMI is one of the most important risk factors for the development of NASH [21,22]. We thus investigated the relevance of BMI for alterations in hepatic fatty acid and glucose metabolism of our pediatric patients (see Figure 2). A high BMI results in increased glycolytic capacity, a characteristic of a (pre)diabetic phenotype (Figure 2a). Additionally, the maximal capacities for the uptake of fatty acids, TAG synthesis, and lipoprotein secretion were positively correlated with the BMI (Figure 2b–d) indicating a general upregulation of lipogenesis. Additionally, we also found a significant association between BMI and alterations in hepatic glucose metabolism.

**Figure 3:**
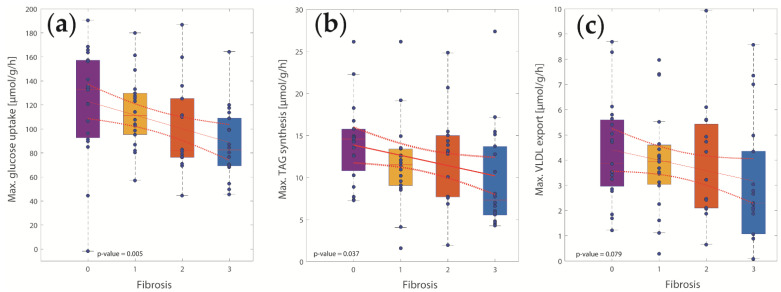
Relationship between histologically assessed liver fibrosis and various hepatic capacities. (**a**) Glycolysis; (**b**) TAG synthesis; (**c**) Lipoprotein production. A high degree of fibrosis is associated with decreased TAG synthesis, reduced glycolysis, and decreased lipoprotein synthesis.

To better understand the metabolic implications of juvenile NASH, we searched for significant associations between metabolic capacities and histologically assessed degree of fibrosis, a key parameter for NASH staging. We found a significant decline in the capacities for TAG synthesis and glucose uptake and an association between decreased lipoprotein synthesis and increasing fibrosis staging (Figure 3).

### 2.3. Concerted Metabolic Alterations in NASH Livers

**Figure 4:**
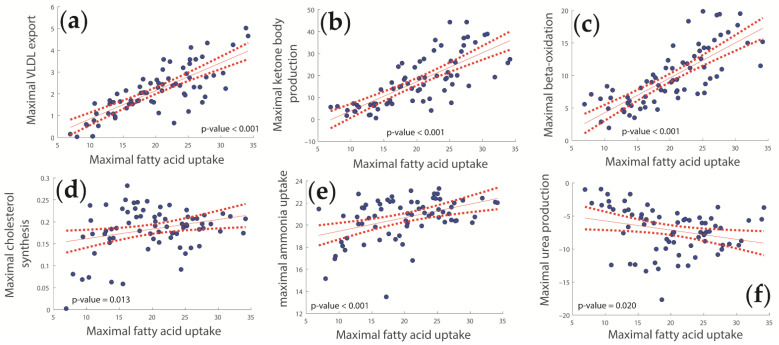
Significant associations between fatty acid uptake and other metabolic functions. (**a**) Lipoprotein synthesis; (**b**) Ketone Body production; (**c**) β-oxidation; (**d**) Cholesterol synthesis; (**e**) Ammonia uptake; (**f**) Urea production. For full information, see Supplemental Figure S1.

The liver must fulfill a multitude of metabolic functions in parallel. Given that the liver of adipose patients is permanently confronted with higher levels of plasma fatty acids, we investigated the relation of various metabolic functions with uptake rates of fatty acids (see Figure 2, Figure 4 and Figure S1). Increasing uptake rates of fatty acids were found to be tightly coupled with increasing rates of lipoprotein synthesis, ketone body production, β-oxidation, and cholesterol synthesis (Figure 4a–d). It is noteworthy that there was no significant correlation of fatty acid uptake with intracellular TAG content (*p* = 0.164), i.e., metabolization of excess free fatty acids by the juvenile NASH liver consists predominantly in their utilization (β-oxidation, export of ketone bodies, and VLDL export) rather than intrahepatic storage.

Intriguingly, with an increasing uptake of fatty acids, the rate of ammonia uptake was also increased, while the rate of urea synthesis was decreased. Thus, at an elevated fatty acid challenge, the liver appears to favor the synthesis of glutamine as the main route of ammonia detoxification.

### 2.4. The Relative Impact of NASH and IR on Alterations of Hepatic Metabolism

The histology-based characterization of the livers showed various degrees of juvenile NASH. In addition, the HOMA-IR indicated varying degrees of IR and pre-diabetes in these children. To decipher the contributions from NASH and IR to hepatic metabolic alterations, we divided the 71 patients into four disease classes of approximately equal size according to the severity (low or high) of NASH and IR. While IR was classified by HOMA-IR, we used two different classification schemes for NASH based either on the degree of inflammation or fibrosis (see Section 4).

**Figure 5:**
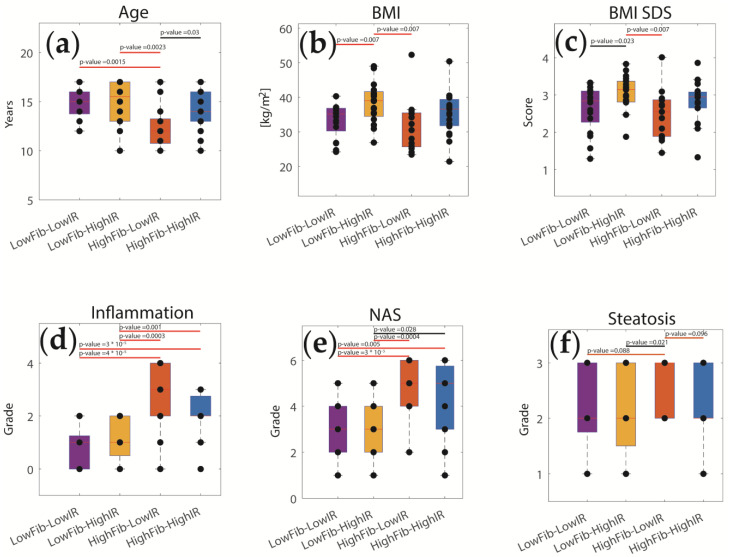
Clinical parameters of pediatric patients in four different disease classes (variant A). (**a**) Age; (**b**) BMI; (**c**) BMI Standard Deviation Score (SDS); (**d**) NAFLD Activity Score (NAS); (**e**) Inflammation score; (**f**) Degree of fibrosis. Crossbars indicate differences between groups based on two-sided *t*-testing (orange: *p* < 0.1; black: *p* < 0.05; red: *p* < 0.01). Corresponding *p*-values are given above bars.

Figure 5 demonstrates significant differences of several clinical parameters between the four classes. For example, patients with high IR had a higher BMI compared to those with low IR, irrespective of the degree of fibrosis. Patients with high fibrosis had significantly higher inflammation (assessed by the NAFLD Activity Score and the inflammation score) compared to those with low fibrosis, irrespective of the HOMA-IR. These findings are in line with the general view that a persistent inflammatory state of the liver leads to fibrosis, and that a high BMI and the accompanying IR of the adipose tissue lead to T2DM.

**Figure 6:**
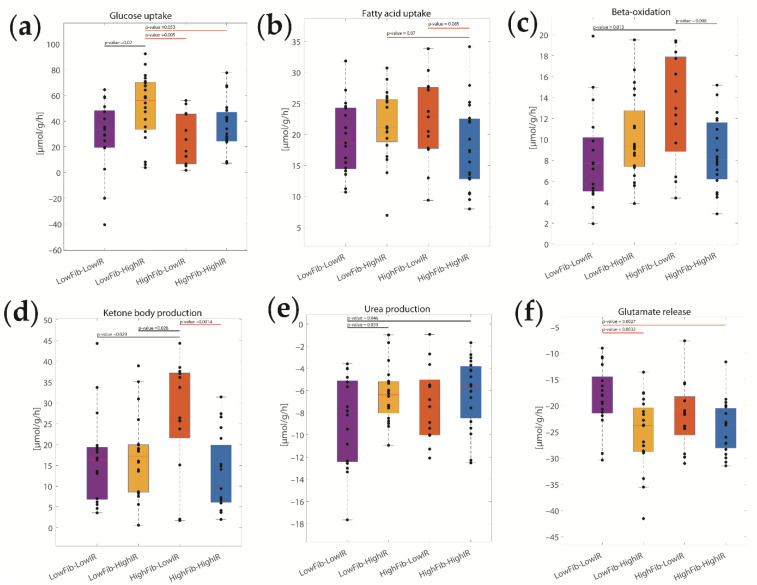
Diurnal differences in metabolic functions between the four disease classes (variant A) defined by the degree of fibrosis and HOMA-IR. (**a**) Rate of glycolysis; (**b**) Fatty acid uptake; (**c**) β-oxidation; (**d**) Ketone body production; (**e**) Urea production; (**f**) Glutamate release. Crossbars indicate differences between groups based on two-sided *t*-testing (orange: *p* < 0.1; black: *p* < 0.05; red: *p* < 0.01). Corresponding *p*-values are given above bars.

We also found significant inter-class differences between several metabolic functions (see Figure 6). For example, the maximal glucose uptake rate was higher in the two classes of patients with high IR, suggesting a higher impact of IR than fibrosis on changes in hepatic glucose metabolism. Significant inter-class differences were also obtained for β-oxidation, the release of ketone bodies, and urea production. Interestingly, no statistically significant differences were found in the uptake of fatty acids, although there was a tendency for decreased fatty acid utilization in patients with both high fibrosis and high IR.

In line with the close association of inflammation and fibrosis in NASH livers, very similar results of this class-based investigation were obtained by evaluating the severity of NASH by the parameter inflammation instead of fibrosis (variant B, see Supplemental Figure S2). Taken together, the metabolic differences among the disease classes pointed to the differential effects of NASH and IR on liver metabolism.

To characterize the association strength of NASH and IR with inter-class differences in metabolic capacities, we used a linear regression analysis (see Section 4). Figure 6 shows the effects of NASH and IR on a wide set of metabolic functions. As can be seen, NASH and IR have opposing impacts on hepatic carbohydrate and lipid metabolism while acting synergistically on ammonia metabolism.

A high value of HOMA-IR is associated with increased hepatic glucose uptake capacity and glycogen content, but both metabolic functions decrease with increasing inflammation or fibrosis. Contrasting to this, fibrosis increases the uptake capacities of β-oxidation and synthesis of ketone bodies, while IR has the opposite effect. In contrast to these opposing effects in carbohydrate and lipid metabolism, fibrosis and IR were both negatively associated with the capacities for ammonia uptake and urea synthesis and positively associated with the capacity for glutamine release. Interestingly, compared to fibrosis, the association strength of metabolic changes with inflammation was generally stronger. This finding suggests that in pediatric patients with NASH, inflammation and fibrosis are both drivers of metabolic changes in the liver, yet some effects may be masked by opposite actions of IR in early metabolic alterations of the liver.

## 3. Discussion

This study aimed to assess changes in liver metabolism in children and adolescents with NAFLD and IR. The direct experimental determination of the metabolic state of the human liver is very limited, as it requires the measurement of arteriovenous concentration differences by catheter techniques and the use of radio-labeled metabolites [23,24]—onerous procedures that can hardly be applied to patients with chronic liver disease, even more so in children. Multinuclear spectroscopy enables the accurate quantification of liver glycogen or TAG content [25] but does not allow for conclusions about changes in the underlying biochemical fluxes. Therefore, we combined our previously developed and validated kinetic models of central liver metabolism (HEPATOKIN1) [14] and lipid droplet metabolism [15] and parameterized the expanded metabolic model for each patient using protein intensity profiles obtained from biopsy samples. With this model, we provide now the most comprehensive and detailed biochemistry-based mathematical model of central liver metabolism.

### 3.1. Association of Metabolic Capacities and Clinical Markers of NASH and IR

To validate our combined proteomics-modeling approach, we first demonstrated the good agreement between calculated and histologically assessed degree of steatosis and computed alterations in hepatic glucose metabolism with HOMA-IR. We identified the upregulation of perilipin 2 as the major factor for the increased TAG storing capacity of the liver. Perilipin 2 is essential for the biogenesis of lipid droplets by affording protection against droplet lipolysis and its upregulation has been reported to result in elevated TAG deposition [26,27]. In adult NASH patients, the synergistic effect of increased de novo lipogenesis, defects of β-oxidation, and reduced export of TAG by VLDL particles have been made responsible for the development of steatosis. Therefore, the transcription factors SREBP1 and ChREBP are key regulators of pro-lipogenic genes [28].

We found significant associations of hepatic fatty acid as well as glucose metabolism with BMI, degree of fibrosis, and age. Consequently, BMI is not only a risk factor for the development of NAFLD but also an important indicator of metabolic changes in the liver of pediatric NAFLD patients. The higher the BMI, the closer the adipose tissue comes to its expansion limit, and the greater the likelihood of developing IR, elevated plasma levels of free fatty acids, and thus the development of NAFLD.

### 3.2. Hepatic Metabolic Alterations in NASH and IR

As NAFLD/NASH and IR/T2DM are closely linked, we examined the collective association of both pathologies by grouping the patients into four disease classes according to the degree of liver fibrosis (variant A) or inflammation (variant B) and the value of HOMA-IR. Our analysis revealed that NASH and IR/diabetes are associated in opposite ways with changes in liver glucose and lipid metabolism, whereas both pathologies act synergistically on increased capacity for glutamine synthesis and reduced capacity for urea synthesis.

As first formulated in 1963 by Randle et al. as a general principle, glucose and free fatty acids are competing substrates of cellular metabolism [29]. This antagonism is not limited to metabolic regulation at the enzyme level but is also realized in the activation of different groups of transcription factors and their associated signaling pathways [30,31]. Whereas steatosis and the resulting NASH are linked to elevated plasma levels of free fatty acids, IR and diabetes are linked to elevated plasma levels of glucose. The competition between these two cardinal metabolites is also manifested in the antagonistic effects on glucose and lipid metabolism in the NASH liver.

### 3.3. Lipid Metabolism

**Figure 7:**
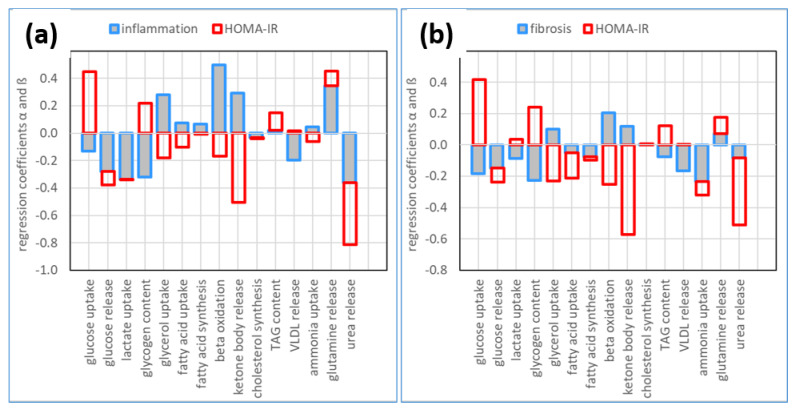
Association strength of NASH and IR with metabolic changes of the liver. The association strength was quantified by the normalized regression coefficients α and β in Equation (1). (**a**) Association strength if the severity of NASH and IR is measured by inflammation grading and HOMA-IR; (**b**) Association strength if the severity of NASH and IR is measured by fibrosis grading and HOMA-IR.

The association of metabolic changes with NASH was higher when the definition of disease classes was based on inflammation and HOMA-IR. Pro-inflammatory cytokines have a strong impact on hepatic lipid metabolism [32,33]. Interleukin 6 and TNFα play a central role in the promotion of liver inflammation and steatosis. We observed an increase in the capacities for β-oxidation and ketone body export, while alterations in TAG content are small (Figure 7). The export of VLDL lipoproteins was even negatively associated with inflammation. In line, a decline of steatosis with increasing inflammation was observed in an animal study with mice, which received a methionine-deficient, choline-deficient diet to induce steatohepatitis [34].

Our analysis provides evidence that ketone body production is oppositely influenced by NASH and IR: IR suppresses the synthesis of ketone bodies, while NASH is associated with increased ketogenesis. While the inhibitory effect of insulin on hepatic ketogenesis (mainly via PPARα) has been well studied [35], little is known about the influence of NASH on ketogenesis. Fletcher et al. found that the synthesis of ketone bodies from free fatty acids was progressively impaired as hepatic steatosis and glycemia worsened [36]. They concluded that the impairment of ketogenesis and increased oxidation of fatty acids in the tricarbonic acid cycle was the reason for increased hepatic glucose production and hyperglycemia. In contrast, we found a significant positive association between steatosis and ketogenesis (see Supplemental Figure S3). Possibly, these conflicting findings can be explained by the fact that the decline of ketogenesis reported by Fletcher et al. [36] was ultimately due to the strong negative effect of hyperglycemia, i.e., hyperglycemia was the actual cause of diminished ketogenesis and not vice versa.

### 3.4. Glucose Metabolism

HOMA-IR was positively associated with hepatic glucose uptake and negatively associated with glucose release, indicating a higher capacity of the liver to lower plasma glucose levels. Fibrosis and inflammation, the two hallmarks of NASH, were negatively associated with both hepatic glucose uptake and release. This indicates that the progression of NAFLD worsens the liver’s contribution to blood glucose homeostasis and thus contributes to the worsening of IR and prediabetes. Concordantly, Cali et al. [37] concluded that the increasing severity of fatty liver in obese adolescent patients is associated with glucose dysregulation. However, if NAFLD is paralleled by IR, the dysregulation of glucose metabolism caused by NAFLD alone appears to be attenuated. This is because juvenile prediabetes in obese adolescent patients with NAFLD improves the function of the liver to remove excess glucose from the blood. It should be emphasized that this scenario holds for our pediatric NASH cohort characterized by increased insulin plasma levels and intact β-cell function. In adult patients with manifest diabetes, i.e., impaired insulin secretion, the situation is entirely different: Deficiency of insulin and relative excess of glucagon increase the expression of key enzymes of gluconeogenesis and regulate interconvertible enzymes toward glucose production, thus converting the liver into a glucose producer similar to under conditions of starvation [38,39].

### 3.5. Ammonia Metabolism

Both NASH and IR were negatively associated with the capacities for ammonia uptake and urea synthesis but positively associated with the capacity for glutamine release. A decline in urea synthesis was reported in both experimental and human NASH and accounted for by the hypermethylation of the promotor of the urea cycle enzyme ornithine transcarbamylase [40]. In our study, downregulation of urea cycle enzymes was observed only in the disease class HighNASH-HighIR, suggesting that inhibition of the urea cycle requires the simultaneous presence of NASH and IR. Possibly, the shift from urea synthesis to glutamine synthesis can be related to the fact that glutamine is a potent activator of fibrosis. Glutaminolysis in hepatic stellate cells has been demonstrated to sustain energy metabolism when quiescent hepatic stellate cells become myofibroblastic [41,42].

### 3.6. Limitations of Our Approach

Our approach was restricted to the assessment of metabolic capacities and functions at fixed concentrations of substrates and hormones. While this is adequate for the evaluation of functional changes due to variable protein abundances, patient-individual variances in circulating nutrients and hormones were not taken into account. Furthermore, we considered hormone-dependent phosphorylation states based on effective transfer functions relating circulating hormone levels to phosphorylation states of interconvertible enzymes, neglecting possible alterations in the underlying signaling cascades that might also differ between patients. Therefore, additional variability regarding circulating nutrients, hormones, and hormonal signaling cascades may impact the metabolic functions of individual livers. We would expect that these additional variabilities would enhance metabolic differences between individual patients.

## 4. Materials and Methods

### 4.1. Study Design and Patients

The study protocol conformed to the guidelines of the Declaration of Helsinki and was approved by the Ethics Committee of the Charité-Universitätsmedizin Berlin (EA2/059/14). Informed consent was obtained from all parents or guardians. Patients were recruited from the pediatric obesity outpatient clinic and pediatric gastroenterology outpatient clinic of the Charité. Blood work including liver function tests was performed as part of standard care and prompted a further workup. Standard serologic tests were obtained for exclusion of α1-antitrypsin deficiency, celiac disease, autoimmune hepatitis, viral hepatitis (type A, B, and C), active cytomegalovirus, or Epstein–Barr virus infection, and Wilson disease. Children and adolescents aged 10 to 17 years who were overweight or obese and suspected of having NASH and a clinical indication for liver biopsy were evaluated for enrollment in the study. Exclusion criteria were as follows: age of at least 18 years, any concurrent liver disease, severe underlying chronic disease (e.g., cardiopulmonary or autoimmune disease), alcohol consumption greater than 20 g per day, and pregnancy. In all patients included in our study, anthropometric measures (height, weight, waist circumference) were taken, and laboratory analysis including a hepatic panel (alanine aminotransferase, aspartate aminotransferase, γ-glutamyltransferase, pseudocholinesterase, and glutamate dehydrogenase) and complete blood count was performed. Metabolic serum parameters including lipid profiles, HOMA-IR, lactate, pyruvate, and uric acid were assessed.

The terms overweight, obesity, and extreme obesity were defined according to guidelines of the German National Work Group as follows: overweight is defined as a body mass index (BMI) above the 90th percentile up to the 97th percentile, obesity as a BMI above the 97th percentile up to the 99.5th percentile, and extreme obesity as a BMI above the 99.5th percentile. Percentiles used are age- and sex-specific and were derived from measurements of height and weight taken in German studies including 17,147 boys and 17,275 girls aged 0 to 18 years [43].

### 4.2. Histological Assessment

All liver biopsies were evaluated and scored by three pathologists (B.R., H.B., and C.L.), each with more than 20 years of experience in hepatic pathology. Discrepancies among pathologists were discussed and a consensus was reached on a final score. Liver biopsies were performed by using a 17-gauge Hepafix liver biopsy set (Braun, Melsungen, Germany). Formalin-fixed, paraffin-embedded biopsy specimens were scored by using 2 µm sections stained with hematoxylin-eosin, chromotrope-aniline blue trichrome, Masson trichrome, and Gomori silver. All biopsy specimens were considered technically adequate for evaluation. Liver biopsies were staged and graded by using the histologic scoring system for NAFLD of the NASH Clinical Research Network [44]. Briefly, grading included the scoring of steatosis (grade 0, <5% of hepatocytes; grade 1, 5–33%; grade 2, 34–66%; grade 3, ≥67%), lobular inflammation (grade 0, no foci per magnification field 200×; grade 1, <2 foci per magnification field 200×; grade 2, 2–4 foci per magnification field 200×; grade 3, >4 foci per magnification field 200×), and hepatocellular ballooning (grade 0, none; grade 1, few ballooned hepatocytes; grade 2, prominent hepatocellular ballooning). The individual scores for each of these three features were summed up to obtain the NAFLD activity score (NAS), ranging from 0 to 8. Moreover, to account for specific features of pediatric NASH, we scored portal inflammation (grade 0, none; grade 1, mild; grade 2, more severe than mild). Five stages of fibrosis were distinguished (stage 0, no fibrosis; stage 1a, delicate peri-sinusoidal fibrosis in zone 3; stage 1b, dense perisinusoidal fibrosis in zone 3; stage 1c, only portal/periportal fibrosis; stage 2, portal/periportal fibrosis and perisinusoidal fibrosis in zone 3; stage 3, bridging fibrosis; stage 4, cirrhosis).

### 4.3. Liquid Chromatography-Mass Spectrometry (MS) Instrument Settings for Shotgun Proteome Profiling and Data Analysis

Liquid chromatography-MS/MS of liver tissue was carried out by nanoflow reverse-phase liquid chromatography (Dionex Ultimate 3000, Thermo Scientific, Waltham, MA, USA) coupled online to a Q-Exactive HF Orbitrap mass spectrometer (Thermo Scientific, Waltham, MA, USA). The LC separation was performed using a PicoFrit analytical column (75 μm ID × 55 cm long, 15 µm Tip ID (New Objectives, Woburn, MA, USA) in-house packed with 3-µm C18 resin (Reprosil-AQ Pur, Dr. Maisch, Ammerbuch-Entringen, Germany) as reported previously [45]. Briefly, peptides were eluted using a gradient from 3.8 to 50% solvent B in solvent A over 121 min at 266 nL per minute flow rate. Solvent A was 0.1% formic acid and solvent B was 79.9% acetonitrile, 20% water, and 0.1% formic acid. Nanoelectrospray was generated by applying 3.5 kV. A cycle of one full Fourier transformation scan mass spectrum (300–1750 m/z, resolution of 60,000 at m/z 200, AGC target 1 × 10^6^) was followed by 12 data-dependent MS/MS scans (resolution of 30,000, AGC target 5 × 10^5^) with a normalized collision energy of 25 eV. To avoid repeated sequencing of the same peptides, a dynamic exclusion window of 30 s was used. In addition, only the peptide charge states between two to eight were sequenced. Raw MS data were processed with MaxQuant software (v1.5.7.4, Max Planck Institute for Biochemistry, Martinsried, Germany) 6 with the Andromeda search engine 7 and the mouse UniProtKB with 51,416 entries released in March 2016. A false discovery rate of 0.01 for proteins and peptides, a minimum peptide length of seven amino acids, a mass tolerance of 4.5 ppm for precursor, and 20 ppm for fragment ions were required. A maximum of two missed cleavages was allowed for the tryptic digest. Cysteine carbamidomethylation was set as a fixed modification, while N-terminal acetylation and methionine oxidation were set as variable modifications.

### 4.4. Computational Assessment of Metabolic Capacities

**Figure 8:**
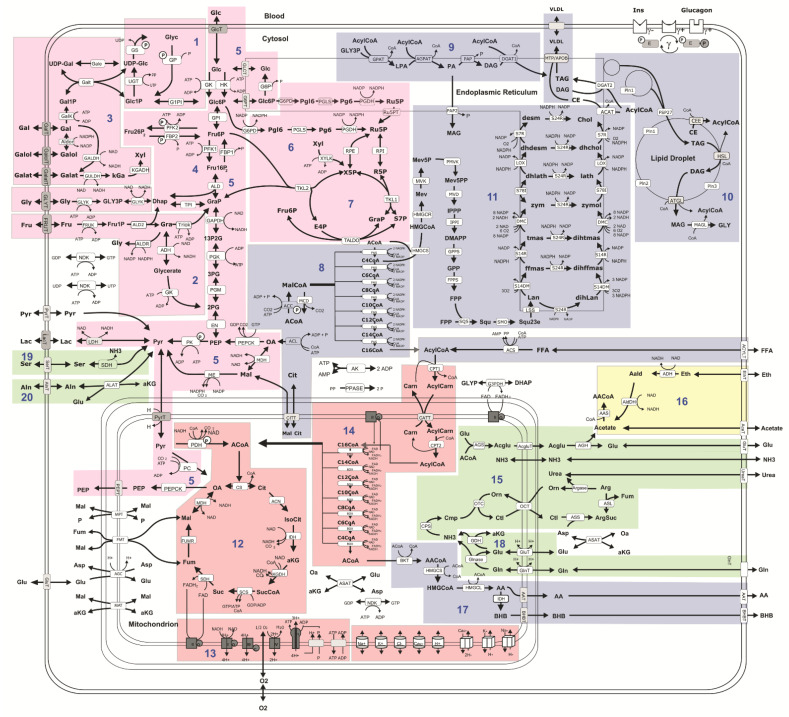
Schematic model representation. Reactions and transport processes between compartments are symbolized by arrows. Single pathways as defined in biochemical textbooks are numbered and highlighted by different coloring: (**1**) glycogen metabolism, (**2**) fructose metabolism, (**3**) galactose metabolism, (**4**) glycolysis, (**5**) gluconeogenesis, (**6**) oxidative pentose phosphate pathway, (**7**) non-oxidative pentose phosphate pathway, (**8**) fatty acid synthesis, (**9**) triglyceride synthesis, (**10**) synthesis and degradation of lipid droplets and synthesis of VLDL lipoprotein, (**11**) cholesterol synthesis, (**12**) tricarbonic acid cycle, (**13**) respiratory chain and oxidative phosphorylation, (**14**) β-oxidation of fatty acids, (**15**) urea cycle, (**16**) ethanol metabolism, (**17**) ketone body synthesis, (**18**) glutamine metabolism, (**19**) serine utilization, and (**20**) alanine utilization. Lipid droplet synthesis and degradation pathways include de novo synthesis of lipid droplets, lipid droplet filling, lipid droplet growth and fusion as well as lipid droplet degradation in dependence on regulatory surface proteins [15]. Small cylinders and cubes symbolize ion channels and ion transporters. Double arrows indicate reversible reactions, which may proceed in both directions according to the value of the thermodynamic equilibrium constant and cellular concentrations of their reactants. Reactions are labeled by the short names of the catalyzing enzyme or membrane transporter given in the small boxes attached to the reactions arrow. Red boxes indicate enzymes that are regulated by hormone-dependent reversible phosphorylation. Metabolites are denoted by their short names. Full names of metabolites and kinetic rate laws of reaction rates are outlined in Berndt et al. [14] and Wallstab et al. [15]. The figure was adapted from Figure 1 of reference [14] by updating and rearranging pathways (**9**)–(**11**) (http://creativecommons.org/licenses/by/4.0/).

Hepatic metabolic capacities were assessed using HEPATOKIN1 [14] in combination with a molecular resolved model of hepatic lipid droplet metabolism [15]. The resulting model is depicted in Figure 8. Hormone-dependent regulation of the liver metabolism by reversible enzyme phosphorylation was taken into account by a phenomenological transfer function [38]. Patient-specific model instantiations were generated based on personal proteomic profiles as described in Berndt et al. [46]. For normalization, seven patients with fibrosis stage F0 and the lowest degree of steatosis S1 were used. Of the 3821 detected proteins, 521 were used for patient-specific modeling corresponding to a coverage of 77.19% ± 8.6 for the metabolic network.

Metabolic functions were defined by the maximal flux obtained using a 24 h standard plasma profile of nutrients and hormones as model input [14]. Metabolic functions for storage of glycogen and TAG were defined by the maximal diurnal concentrations (see Supplemental Figure S1). Maximal metabolic capacities were assessed as maximal flux values under physiological conditions, where plasma metabolite concentrations are not independent of each other. Under physiological conditions, glucose stimulates insulin release from beta cells, concomitant reduces glucagon release from alpha cells in the pancreas, and both hormones control the release of fatty acids from adipose tissue. The interdependence between plasma glucose, plasma hormone, and plasma fatty acid concentration was considered using the sigmoid Hill-type function, describing the experimentally determined glucose–insulin, glucose–glucagon, and glucose–fatty acid relations [14,15,38]. Variations of plasma glucose levels were performed for various physiological conditions, ranging from prolonged fasting (3 mM) to excessive nutrient uptake (12 mM plasma glucose).

### 4.5. Disease Classes Based on the Severity of NNASH and IR

The 71 patients were divided into four disease classes of approximately equal size according to the severity of NASH and IR. Homeostasis model assessment for insulin resistance (HOMA-IR) was used as a biomarker for the severity of IR [18]. For the classification of NASH activity, we stratified patients with regard to fibrosis stage (Fib; variant A) and grade of inflammation (Inf; variant B). The cut-off values for a low or high severity were for variant A: class LowFib-LowIR: fibrosis < F2 and HOMA-IR < 5.5; class LowFib-HighIR: fibrosis < F2 and HOMA-IR ≥ 5.5; class HighFib-LowIR: fibrosis ≥ F2 and HOMA-IR < 5.5; class HighFib-HighIR: fibrosis ≥ F2 and HOMA-IR ≥> 5.5; and for variant B: LowInf-LowIR: inflammation < 2 and HOMA-IR < 5.5; class LowInf-HighIR: inflammation < 2 and HOMA-IR ≥ 5.5; class HighInf-LowIR: inflammation ≥ 2 and HOMA-IR < 5.5; class HighInf-HighIR: inflammation ≥ 2 and HOMA-IR ≥ 5.5. The severity of inflammation was scored by adding the scores for portal inflammation (0–2) and lobular inflammation (0–2).

To characterize the association strength of NASH and IR with inter-class differences in metabolic capacities, we used a linear regression function:

M_i_ = Mo + α × (inflammation/fibrosis)_i_ + β × (HOMA-IR)_i_ (1)

relating the average metabolic capacity M_i_ of disease class i to the average grade of fibrosis and HOMA-IR. The magnitude and sign of the regression coefficients α and β were taken to quantify the relative strength of association of NASH and IR with the respective metabolic capacity. To make the values of α and β comparable for different metabolic capacities, the values for the variables M, inflammation (ranging from 0 to 4), fibrosis (ranging from 0 to 3), and HOMA-IR (ranging from 1.3 to 21) were scaled to the interval [0,1].

### 4.6. Statistics

Two-sample association testing was based on Pearson’s correlation coefficient with *p* < 0.05, indicating statistical significance.

## 5. Conclusions

Children and adolescents with NAFLD already show significant alterations in liver metabolism that increase with the progression of NASH. Steatosis, the initial stage in NAFLD development, is strongly coupled to changes in the lipid droplet turnover of hepatocytes. NASH and IR, two closely linked but different disease patterns, display both synergistic and antagonistic associations with metabolic changes in the liver, and their intrinsic co-occurrence can mask early metabolic alterations in the liver.

## Figures and Tables

**Table 1:** Selected clinical parameters highlighting general features of NAFLD in the study group. Values are expressed as mean ± SD unless indicated otherwise. SD = standard deviation; BMI = body mass index; BMI z-score = body mass index standard deviation score adjusted for age and sex; CRP = C-reactive protein; ALT = alanine aminotransferase; AST = aspartate aminotransferase; HOMA-IR = homeostatic model assessment for insulin resistance; HbA1c = hemoglobin A1c.

Variable	Mean ± SD
Age (years)	14.2 (11–17)
Male sex, *n* (%)	55 (77.5)
BMI	34.9 ± 6.4
BMI z-score	2.77 ± 0.59
CRP (mg/L)	4.7 ± 6.6
ALT (U/L)	108 ± 68
AST (U/L)	63 ± 41
Triacylglycerol (mg/dL)	136 ± 72
Total cholesterol (mg/dL)	165 ± 29
HOMA-IR	7.0 ± 3.7
HbA1c (%)	5.4 ± 1.0

**Table 2:** Histologic evaluation of NAFLD in the study group. Numbers in brackets represent frequencies (%) of histologic findings in the study population.

Grade/Stage	Steatosis	Portal Inflammation	Lobular Inflammation	Ballooning	Fibrosis
0	-	26 (36.6)	22 (31.0)	37 (52.1)	19 (26.8)
1	13 (18.3)	37 (52.1)	39 (54.9)	27 (38.0)	19 (26.8)
2	25 (35.2)	8 (11.3)	10 (14.1)	7 (9.9)	15 (21.1)
3	33 (46.5)	-	-	-	18 (25.4)

## Data Availability

The data presented in this study are available on request from the corresponding author.

## Supplementary Materials

The following supporting information can be downloaded at: https://www.mdpi.com/article/10.3390/ijms231911072/s1.
