# Supplementary material for: Alterations of Central Liver Metabolism of Pediatric Patients with Non-Alcoholic Fatty Liver Disease
Source: Int J Mol Sci. 2022 Sep 21;23(19):11072. doi: 10.3390/ijms231911072 (PMC9570193; doi:10.3390/ijms231911072)
Supplement: Supplementary file 1 [file ijms-23-11072-s001.zip › ijms-1903187-supplementary.pdf]

Supplemental Figures

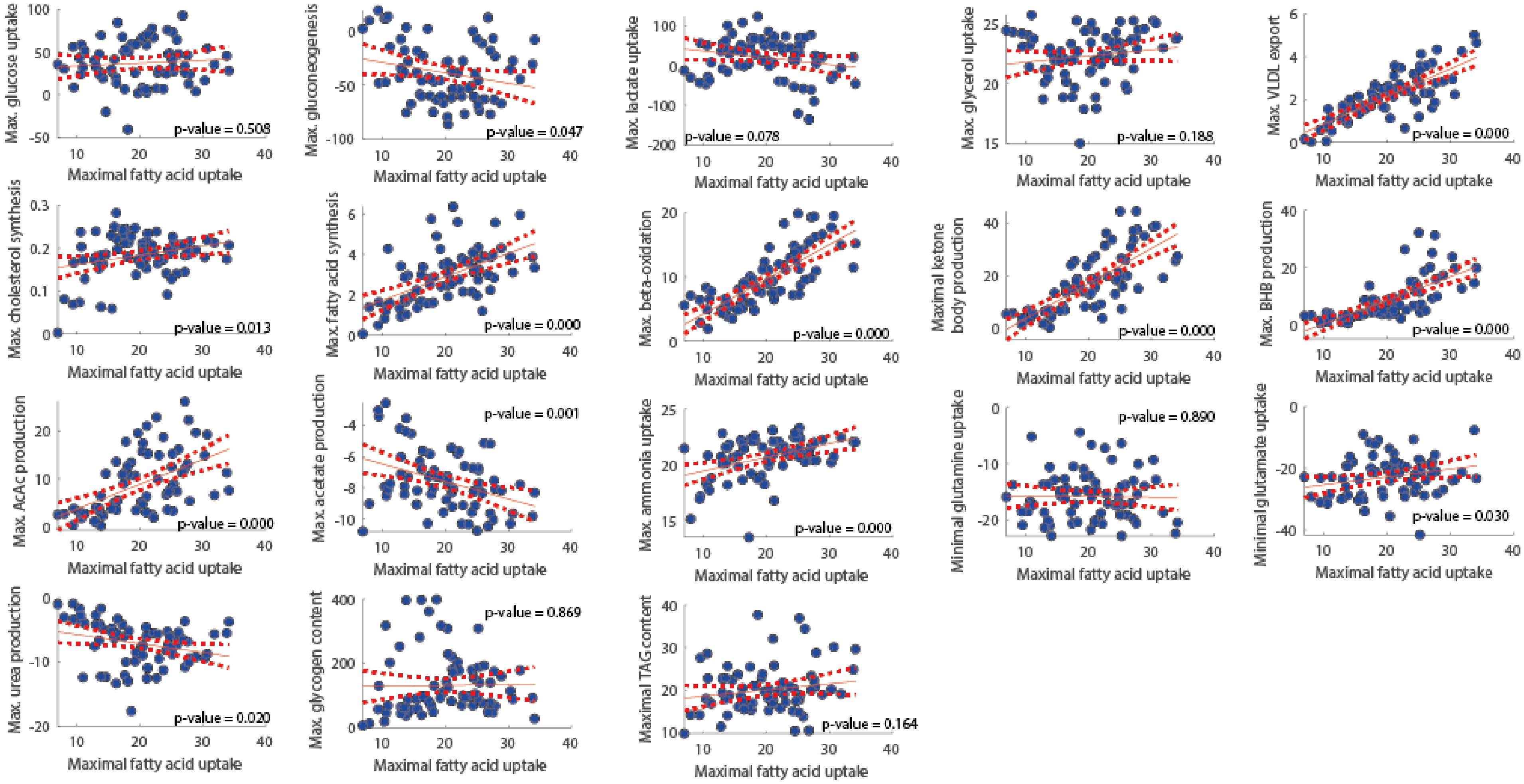

Supplemental Figure S1 Associations between fatty acid uptake and other metabolic functions. AcAc: acetoacetate; BHB: β-hydroxybutyrat; TAG: Triacylglycerol; VLDL: Very low density lipoprotein.

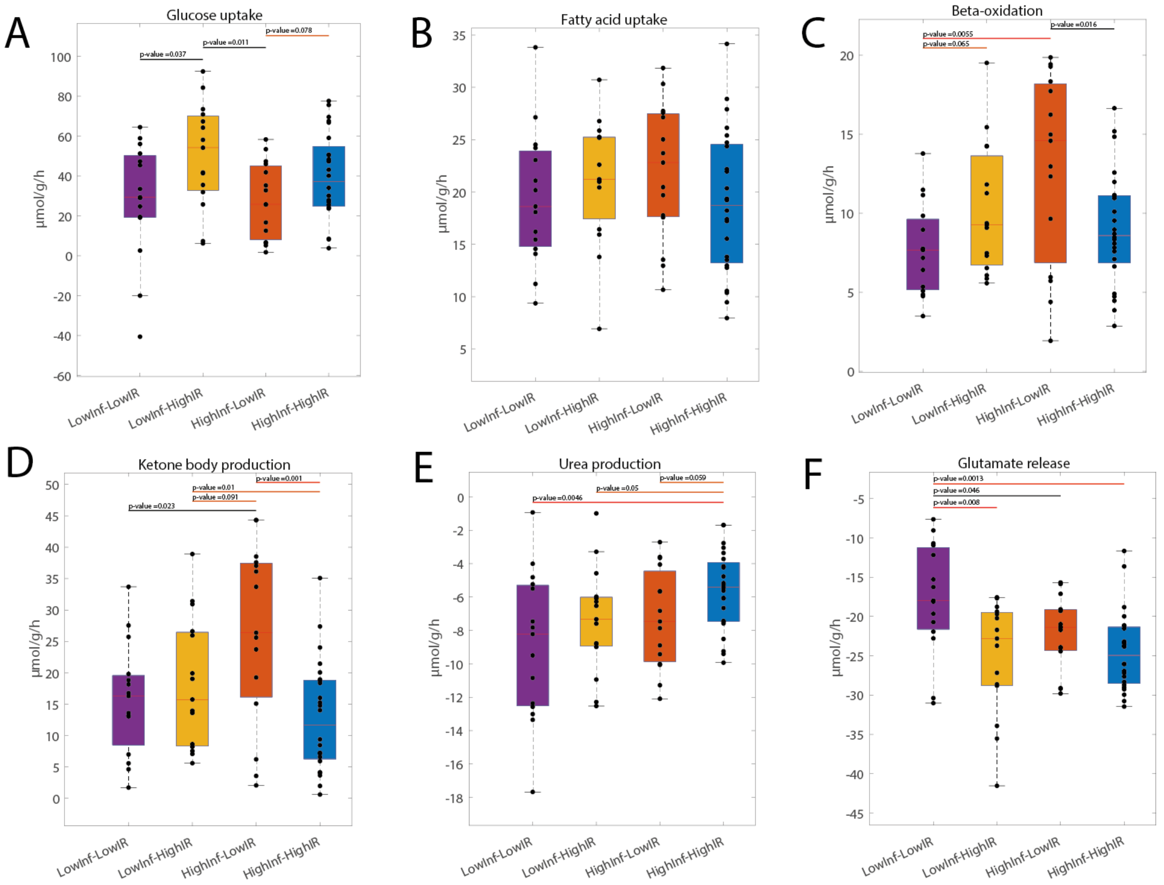

Supplemental Figure S2 Diurnal differences in metabolic functions between four disease classes defined by the degree of inflammation and HOMA-IR (variant B). (A) Rate of glycolysis; (B) Fatty acid uptake; (C) β-oxidation; (D) Ketone body production; (E) Urea production; (F) Glutamate release. Bars indicate differences between groups based on two-sided t-testing. Corresponding *p*-values are given above bars.

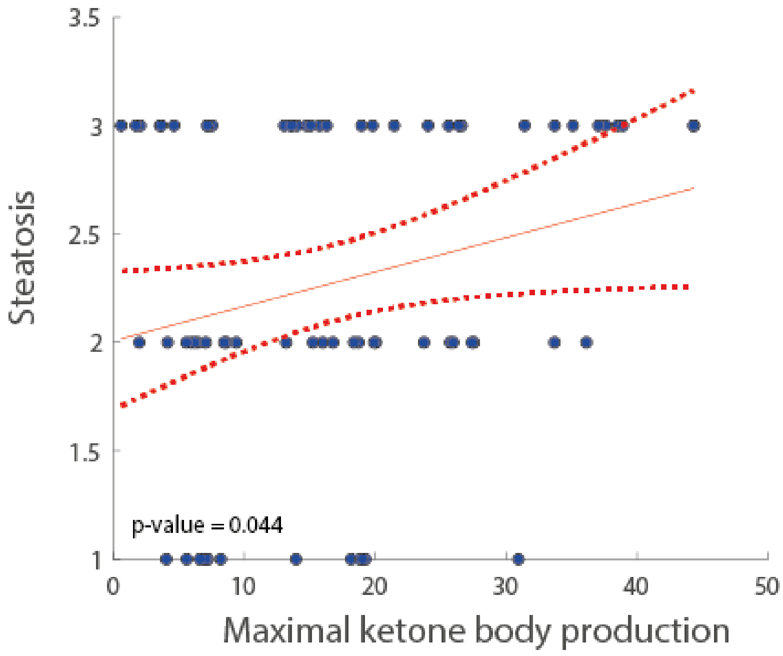

Supplemental Figure S3 Relationship between maximal ketone body production and histologically assessed grade of steatosis.
